# The role of data-driven services strategy in platform competition: A system performance perspective

**DOI:** 10.1371/journal.pone.0272547

**Published:** 2023-01-26

**Authors:** Qiang Hu, Jiaping Xie, Guangsi Zhang

**Affiliations:** 1 College of Business, Shanghai University of Finance and Economics, Shanghai, China; 2 Shanghai University of Finance and Economics Zhejiang College, Jinhua, China; 3 School of Business Administration, Xinjiang University of Finance and Economics, Urumqi, China; URV: Universitat Rovira i Virgili, SPAIN

## Abstract

In the era of big data, data-driven services (DDS) have become critical competitive strategies for digital platform-based enterprises. This paper considers two operational modes of e-commerce platforms, which are self-operated and third-party modes, respectively, and they each lead a platform system. The Hotelling model is adopted to describe the competitive market of both platforms. We characterize their system performance functions. The optimization models are built using game theory to discuss the DDS and price decisions. We obtain the implementation conditions of DDS strategies for both platforms and the dominant situations of their respective DDS levels. We find that a platform adopting the price reduction strategy can improve the performance of its platform system while reducing the competitor’s system performance. From the system performance perspective, continuous improvement of the DDS level may appear “harming others may not benefit oneself”; that is, continuously improving the DDS level leads to a decrease in the competitor’s system performance but not necessarily an increase in its system performance. Further, consumer welfare within both platform systems shows the law of “as one falls then another rises”. As the big data industry matures, self-operated platforms would demonstrate the advantages of service level, profit, and system performance. In contrast, third-party platforms would have an advantage in consumer welfare. These conclusions have important implications for e-commerce platforms developing data-driven operations-based strategies.

## 1. Introduction

DDS (Data-driven services) refers to the services providers adopting big data technology to collect and analyze users’ data to provide higher quality services. In a way, the DDS level represents service providers’ big data analysis and application capabilities. As early as 2011, data-driven business analysis was identified as one of the four major technology trends in the IBM Tech Trends Survey [1]. The report “IDC FutureScape: Worldwide Digital Transformation 2021 Predictions” (Doc # US46880818) released by the International Data Corporation (IDC) shows that by 2025, driven by the turbulent global environment, 75% of enterprise leaders would use digital platforms and ecosystem capabilities to adjust their value chains to adapt to new markets. With the help of big data analysis, enterprise managers can improve business decision-making and performance [2]. In China, leading e-commerce platforms have continued to invest in the field of big data technology in recent years, such as Alibaba and JD.com, which have become technologically innovative companies that provide high-tech services for consumers. E-commerce platforms have large amounts of demand-side data (including clickstreams, transactions, reviews, and other data). Compared with traditional offline retailers, e-commerce platforms have an advantage in collecting, storing, and analyzing abundant data. They can provide consumers with high-quality services in terms of intelligent customer service, accurate recommendation, platform UI design, return and exchange procedures, and selection of the logistics service provider. Therefore, implementing DDS strategies on e-commerce platforms can improve the consumers shopping experience and generate added value for consumers.

A platform ecosystem is a collaborative system in which various cooperative entities use information technology to achieve open mesh contact under the platform’s leadership, which attracts participating enterprises (ecosystem complementors) to enter and jointly provide users with products and services [3]. The research on platform ecosystems has the characteristics of multidisciplinary integration, covering service management, information management and management science, and other fields. Among them, the governance decisions of platform owners and the strategic issues of platform complementors have attracted much attention [4]. In the value co-creation process of platform ecosystems, the coordination between platforms and participants (including users) is of great significance, rather than platforms only pursuing profit maximization. As the ecosystem leader, the platform needs to establish a collective identity for members within the ecosystem, co-supporting the provision of DDS that leads to benefits for the leader and the ecosystem complementors. Note that, in a third-party e-commerce platform ecosystem, the e-commerce platform, the merchant, and consumers are the key system members. Promoting the ecosystem’s value co-creation is the platform leader’s primary task [5].

These lead us to think about how e-commerce platforms implement DDS strategies to improve the performance of their platform systems, especially in a competitive environment. Moreover, there are different operational modes of e-commerce platforms in the market, and the typical ones are self-operated and third-party modes. Therefore, what are the differences in the implementation of DDS strategies for e-commerce platforms with different operation modes, and how do quantify and evaluate the performance of platform systems? In summary, we pay particular attention to the following research questions:

*RQ1*. What are the conditions for e-commerce platforms to implement DDS strategies in a competitive market?
*RQ2*. Whether the implementation of DDS strategies is affected by the operational modes of e-commerce platforms (self-operated or third-party modes)?
*RQ3*. What’s the impact of DDS strategies on platforms’ profits, consumer welfare, and the performance of platform systems?

In order to answer these research questions, we consider a duopoly market consisting of two competing e-commerce platforms, which are self-operated and third-party modes, respectively. E-commerce platform implements DDS strategies, that is, strategic investment in the field of big data to improve their services’ competitiveness. The Hotelling model is used to describe consumer demand, and the optimization model is constructed based on game theory. The conclusions of this paper are obtained by analyzing the equilibrium results of the model. In summary, our research produces four key contributions. First, we have derived the conditions and optimal decisions for these two e-commerce platforms to implement DDS strategies. Second, we measure the system’s performance of self-operated and third-party e-commerce platforms. Third, we find impacts of the implementation of DDS strategies on platform profits, consumer welfare, and platform systems performance. Fourth, we propose useful suggestions for implementing DDS strategies for e-commerce platforms based on the perspective of platform systems performance. We also provide managerial insights for the construction of platform ecosystems. The research has practical value for platform-based enterprises to develop data-driven operations strategies in the context of the digital era.

The rest of this paper is organized as follows. Section 2 reviews the relevant literature. Section 3 describes the research model and makes the basic assumptions. Section 4 explains the sequence of events, solves the model, and analyzes the equilibrium results. Section 5 compares and analyzes the system performance of the two e-commerce platforms. Section 6 contains numerical cases. Section 7 summarizes the conclusions and managerial insights presented in this paper. All the proofs are shown in the S1 Appendix.

## 2. Literature review

This paper is relevant to three streams of the literature, one is the E-commerce platform and its operational management, the other one is platform ecosystems, and the last one is data-driven operations management.

### 2.1 E-commerce platform and its operational management

With the development of big data and new information technology, various types of digital platforms have emerged in recent years, such as online sales platforms, short video platforms, sharing platforms, and freelance platforms [6, 7]. More and more scholars have begun to pay attention to the issue of platform operation and management [8–11]. Similar to other online platforms, e-commerce platforms attract merchants and consumers to join them for online transactions, and the platform charges merchants or consumers a commission to obtain profits [12]. The current research on e-commerce platforms mainly includes platform pricing [13], information matching and security [14, 15], online reviews [16, 17], logistics [18], competition [19], and other issues. Dou et al. [13] held that reasonable pricing could help improve users’ utility on two sides of the platform. Long et al. [14] studied the combination of private information about products held by merchants and private information of consumers held by the platform for advertising auctions to maximize market profits. Jamra et al. [15] advocated that stakeholders should improve the security of e-commerce platforms to avoid property damage to consumers. Chevalier et al. [16] held that online reviews might influence the purchase intention of other consumers on the platform. Saia et al. [17] helped advertisers segment users by analyzing user semantics. Wang et al. [18] demonstrated the heterogeneity in consumers’ participation patterns of value co-creation in e-commerce last-mile logistics. Lai et al. [19] found that there may be a competitive relationship between the third-party merchants on the platform and the platform’s self-operated sales; under certain conditions, the two can achieve simultaneous benefits. Although there have been many aspects of research on e-commerce platforms, there are no related studies on the competition between self-operated and third-party platforms as far as we know, especially considering both horizontal and vertical games.

### 2.2 Platform ecosystems

More and more scholars pay attention to platform ecosystems rather than just considering the short-run profit maximization of the platform [20]. Effective governance of the platform ecosystem is crucial to the coordinated and sustainable development between the platform and its system members [21, 22]. Platform ecosystems can be conceptualized as meta-organizations or “organizations of organizations” that are less hierarchical and less formal than firms yet more closely coupled than traditional markets [23]. Foerderer [4] emphasized the importance of complementary innovation among members within a platform ecosystem through an empirical analysis of companies participating in Apple’s WWDC 2016. E-commerce platform systems usually contain members such as platforms, merchants, and consumers. Panico and Cennamo [24] discussed how user preferences for ecosystem innovativeness and ecosystem size shape the strategic interactions between a platform provider and complementors in the platform ecosystem. Hein et al. [25] highlighted that B2B e-commerce platforms follow three standardized value co-creation practices. Though scholars are increasingly concerned about platform ecosystems, little research has been done on the ecosystemic issues of heterogeneous platforms with competitive relationships. Besides, current research on platform ecosystems mostly uses empirical studies or qualitative research methods (e.g., [4, 23, 25]); however, this paper quantifies the performance of platform ecosystems by drawing on microeconomic theory.

### 2.3 Data-driven operations management

By leveraging the capability of digital technologies, platforms have competitive advantages over traditional businesses [26]. Increasingly, e-commerce platforms are looking to technology-based tools to help merchants engage consumers and enhance the user experience [27]. Therefore, the academic community is beginning to focus on DDS [28–30], data-driven decision-making [31], data-driven performance [32], and the big data discriminatory pricing on Internet platforms [33]. Reviewed the research on data-driven retail operations in the field of operations research/management science in recent years. The research on data-driven is developing rapidly [28]. Thanks to the availability of high-quality data and the advancement of big data technology. Information technology is indispensable to data-driven operations. Saleem et al. [29] explained the strategic data-driven approach for improving business sales performance and conversion rates of e-commerce websites. Kaiser et al. [30] conducted an empirical analysis study on DDS through the data generated during the use of vehicles and interviews with 11 well-known experts in the automotive industry of China and Europe. Wagner et al. [31] found that the distribution of free DDS providers may not be fair and believed that users should analyze privacy risks and benefits when deciding to trade with them. Gawankar et al. [32] concluded through empirical analysis of sales in India that using data technology to process consumer data can help companies improve service levels and enhance the consumers shopping experience. Furthermore, Liu et al. [6] found that online retail platforms have obvious data advantages since they own sales, clickstreams, competitors’ information, and other data. Sharing this big data with merchants can help them make better sales decisions, and the platform will also make profits so that big data can drive marketing. The conditions for implementing DDS strategies have been studied but unclear (e.g., [29, 31]), and the impacts of the DDS strategy on all parties of the platform are not quantified. Thus, whether DDS will affect the competition between self-operated and third-party platforms is worth studying.

In summary, the above studies affirm the business value of data. Platform-based enterprises can use big data technology to make operational decisions such as improving service levels [29]. Besides, big data plays a positive role in platform ecosystems governance. The previous literature provides a reference for studying DDS strategies in this paper. Clearly, the implementation of DDS strategies requires significant investment [32]. Faced with this fact, however, lack of literature on considering the conditions and optimal decision-making behavior of operating model heterogeneous e-commerce platforms to implement DDS strategies, especially in a competitive environment. In addition, most of the previous studies related to platform ecosystems use empirical research or case study methods [4, 23, 25], there is a lack of quantitative evaluation concerning the overall performance of platform systems, and a lack of literature on a quantitative study of the platform systems performance by constructing mathematical models. For these reasons, this paper makes a supplementary study that enriches the theory of data-driven operations management and ecosystems governance in the field of e-commerce.

## 3. Model description and basic assumptions

### 3.1 Two e-commerce platforms

This paper considers two competing e-commerce platforms, one is a self-operated platform (denoted by *S*), and the other is a third-party platform (denoted by *T*). In practice, a self-operated platform such as JD.com and a third-party platform such as Tmall.com, these two modes can be considered integrated and independent systems, respectively [34]. We adopt the Hotelling model [35], which is a classic model to study the differentiated competition of two sellers in the same market [36]. We consider that platforms *S* and *T* share a linear sales market with length 1. Platform *S* is located on the 0-side, and platform *T* is located on the 1-side. Note that the products sold by both platforms have the same material function. In the self-operated mode, platform *S* sells products to consumers at a retail price; we let *c*_*s*_ denote the unit cost of product sales (including product procurement cost, operating cost, etc.). In the third-party mode, the main body of product sales is the settled merchant who determines the product’s retail price *p*_*T*_ on platform *T*. Then, platform *T* charges the merchant a sales commission rate *λ*. In reality, Tmall.com charges its settled merchants a commission rate ranging from 3% to 5% of sales depending on different product categories; the commission rate for most SKU (stock keeping unit, that is, minimum inventory unit) products in JD.com’s independent system is between 2% and 10% [34].

**Fig 1:**
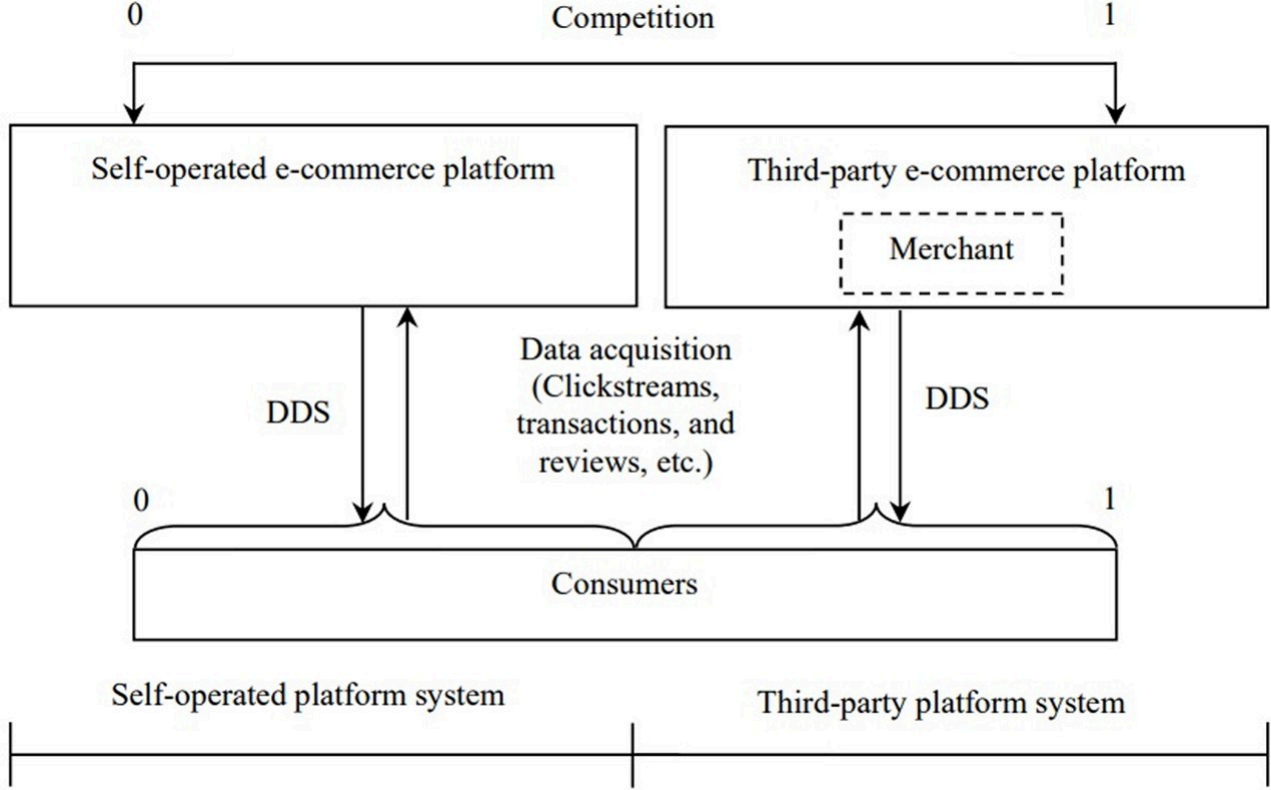
The framework for e-commerce platforms to implement DDS strategies.

The e-commerce platform implements the DDS strategy, which uses big data technology to collect and analyze user data such as consumer clickstreams, transactions, and reviews to understand consumer preferences better and ultimately provide accurate service for consumers. Data can drive marketing and improve consumer utility [6], and it can also help enterprises improve product sales [37]. Fig 1 shows the basic framework for implementing DDS strategies.

Of course, the implementation of a DDS strategy requires big data technology investments of irreversible nature. In this paper, we consider that two e-commerce platforms invest in the field of big data technology to improve DDS levels in order to win consumers’ favor and realize value co-creation of platform ecosystems. In addition to using big data technology to improve the level of DDS in e-commerce platforms, other platform-based enterprises have also used this technology. For example, Ctrip.com uses it to help consumers snatch train tickets and provide consumers with a variety of schemes for snatching tickets, including transfer, obtaining more stations (i.e., earlier than the actual boarding station or later than the actual destination station) and fewer stations (i.e., pay upon arrival), etc. These help to improve the success rate of ticket-snatching, which reflects the high-quality service level. The variable *d*_*i*_ indicates the DDS level of the e-commerce platform, and *i* = *S*, *T* represent platforms *S* and *T*, respectively. Correspondingly, the e-commerce platform needs to pay the investment cost of $I_{i}(d_{i})=kd_{i}{}^{2}/2$; this cost function is increasing at an increasing rate, which has been widely adopted in previous literature [13, 38, 39]. Note that this equation expresses the law of marginal increase in the investment cost of e-commerce platforms with the improvement of DDS level, where *k* represents the influence coefficient of DDS level on investment cost. Specifically, the value of *k* reflects the current situation of big data technology required to improve the DDS level. In other words, a smaller value of *k* indicates that the big data industry is relatively mature, and a larger value of *k* indicates relative industrial backwardness. Let *π*_*i*_ denote the profit of the e-commerce platform.

### 3.2 The merchant

In the self-operated mode, platform *S* is a retailer selling products directly to consumers. Therefore, we do not consider the merchant as a member of the platform system. In the third-party mode, the merchant is the subject of the sale on platform *T* and determines the retail price *p*_*T*_ to consumers. This merchant may be a product manufacturer or a product distributor. Depending on the settlement contract, the merchant must pay a sales commission to platform *T*. Clearly, the merchant is included in the third-party platform system. Note that a merchant can sell on both platforms simultaneously, only in different ways of cooperation. In this paper, we consider that this merchant is not a participant in the self-operated platform system, but a participant in the third-party platform system. Finally, the profit of the merchant on platform *T* is denoted by *π*_*M*_, and the unit sales cost of the merchant (including production or wholesale, operation costs, etc.) is denoted by *c*_*M*_.

### 3.3 Consumers

The Hotelling model is used to describe consumers’ selection of both platforms. Assuming that the population of consumers in the market is 1 and uniformly distributed in the [0,1] interval, the distribution density is 1 [40]. The initial utility that consumers can obtain by buying per unit product on both platforms is *v*, which is large enough to make the two platforms cover all consumers; each consumer selects one of both platforms to maximize their utility; that is, the total market share of both platforms is 1 [41]. Let *x* be the distance from the consumer to platform *S*, *tx* is the travel cost of consumers at point *x* to buy products from platform *S*, and *t*(1 - *x*) is the travel cost when purchasing from platform *T*, where *t* is the unit travel cost in the classical Hotelling model, and obviously *x* ~ *U*(0,1). We remark that the travel cost of consumers entering the two e-commerce platforms for shopping is sufficiently low (even negligible) in the context of the Internet. Therefore, we interpret the location differences as heterogeneity of the two e-commerce platforms, which makes the Hotelling model more consistent with the research situation of this paper. Note that the travel cost can be considered as the “preference cost” of consumers to both platforms; *t* represents the degree of differentiation between both platforms; the smaller *t* is, the less significant the difference between both platforms is, which has also been extended to the research on Internet platform-based operations management by some scholars [11, 41–43]. More importantly, this extension is consistent with the idea proposed in this paper that e-commerce platforms implement DDS strategies to meet consumer preferences better.

Since e-commerce platforms implement DDS strategies to meet consumer preferences in terms of intelligent customer service, accurate recommendation, platform UI design, return and exchange procedures, and logistics provision, so that consumers have a better shopping experience, therefore, consumers can obtain additional utility under DDS strategies. We assume that ${\hat{u}}_{i}$ is a function with respect to *d*_*i*_, and ${\hat{u}}_{i}$ represents the additional utility available to a consumer at the DDS level *d*_*i*_. Furthermore, we consider that ${\hat{u}}_{i}$ is increasing linearly with the improvement of the DDS level, that is, ${\hat{u}}_{i}=hd_{i}$ (when [44] considered that improving the platform’s performance could increase consumer utility and [6] assumed that data-driven marketing could improve consumer utility, they also characterized the relationship as a linearly increasing function), where *h* is the sensitivity coefficient of DDS level to the additional utility; in other words, the value of *h* represents consumer sensitivity to the quality of platform services. In reality, however, e-commerce platforms will not endlessly invest in DDS to attract all consumers. Therefore, we consider setting $h\in (0,2\sqrt{kt})$ mainly based on the following two considerations: on the one hand, it means that the value of *h* is not so large that all consumers enter only one platform; on the other hand, this condition ensures that there exist internally optimal solutions for the optimization problem of this paper, note that [41, 45] have also provided a similar approach.

A summary of the notations used in this paper is provided in Table 1.

**Table 1:** Notations and descriptions for the model.

Notations	Descriptions
**Subscript**	
*i*	*i* = {*S*, *T*} represent self-operated and third-party platforms, respectively
**Superscript**	
*	Market equilibrium
**Decision variables**	
*p*_*i*_	The retail price of the product on platform *i*; where *p*_*s*_ is the decision variable of platform *S*, and *p*_*T*_ is the decision variable of the merchant settled on platform *T*
*d*_*i*_	Platform *i*’s DDS level
**Parameters**	
*λ*	The commission rate charged by platform *T* to the merchant
*k*	The influence coefficient of DDS level on investment cost
*v*	A consumer’s initial utility when buying from both platforms
*h*	The sensitivity coefficient of DDS level to a consumer’s additional utility
*x*	The distance from consumers to platform *S*, *x ~ U(0*,*1)*
*x*_0_	The utility indifference point, meaning that consumers located at this point are indifferent about buying from platform *S* or platform *T*
*t*	Unit preference cost, also called switching cost, represents the degree of differentiation between the two platforms
*c*_*s*_	Unit product sales cost of platform *S*
*c*_*M*_	Unit product sales cost of the merchant settled on platform *T*
**Functions**	
${\hat{u}}_{i}$	The additional utility derived from platform *i* under the DDS strategy
*I*_*i*_	The DDS investment cost of platform *i*
*u*_*i*_	A consumer’s utility when buying from platform *i*
*D*_*i*_	Market demand of platform *i*
*π*_*i*_	Profit of platform *i*
*π*_*M*_	Profit of the merchant settled on platform *T*
*CW*_*i*_	Consumer welfare on platform *i*
*U*_*i*_	System performance of platform *i*

## 4. Model analysis

### 4.1 Sequence of events

Both horizontal and vertical games are present in our model. The term “horizontal game” describes the competition between the self-operated platform and the third-party platform regarding the retail price *p*_*i*_ and DDS level; we consider it a duopoly market competition [11, 42, 43]. The term “vertical game” describes the competition between the third-party platform and the merchant, we consider it as a Stackelberg game [46], and the third-party platform is the Stackelberg leader; that is, the third-party platform first determines the DDS level *d*_*T*_, and the merchant then determines the retail price *p*_*T*_. In the game, each stakeholder makes decisions based on profit or utility (consumers) maximization; the time line of the game is as follows:

*Stage 1 (Horizontal game)*: platform *S* determines the DDS level *d*_*S*_ and the retail price *p*_*S*_, and platform *T* determines the DDS level *d*_*T*_ simultaneously.
*Stage 2 (Vertical game)*: at the given DDS level *d*_*T*_, the merchant settled on platform *T* determines the retail price *p*_*T*_.
*Stage 3 (Consumer purchase decision)*: consumers choose one of both platforms to make a purchase based on utility maximization.

Note that although the price decision on the third-party platform takes place in Stage 2, we assume that the retail price of the product on both platforms can be adjusted simultaneously because the third-party platform is the leader of the vertical game. It can determine the retail price of the merchant by changing the DDS level *d*_*T*_. This assumption makes the horizontal game act simultaneously (the way Chakraborty et al. [46] articulated the sequence of events is instructive).

### 4.2 The demand

At Stage 3, we can derive the product market demand of both platforms. According to the model described in Section 3, the utilities of a consumer located at point *x* when buying a unit product from platform *S* and platform *T* can be expressed as the following equation set:

$$\left\{\begin{array}{l} u_{S}=v-p_{S}-tx+{\hat{u}}_{S}(d_{S})\\ u_{T}=v-p_{T}-t(1-x)+{\hat{u}}_{T}(d_{T}) \end{array}\right.$$ (1)

From Eq (1), the utility indifference point where a consumer’s utility of the two alternatives is equal when buying from both platforms can be obtained as follows:

$$x_{0}=\frac{p_{T}-p_{S}+t+h(d_{S}-d_{T})}{2t}$$ (2)

Thus, the market demand functions of both platforms are shown as follows:

$$\left\{\begin{array}{l} D_{S}(p_{S},p_{T},d_{S},d_{T})=x_{0}=\frac{p_{T}-p_{S}+t+h(d_{S}-d_{T})}{2t}\\ D_{T}(p_{S},p_{T},d_{S},d_{T})=1-x_{0}=\frac{p_{S}-p_{T}+t-h(d_{S}-d_{T})}{2t} \end{array}\right.$$ (3)

### 4.3 Model solving and equilibrium analysis

At Stage 2, we can solve the vertical game model. First, the merchant’s optimization problem can be formulated by:

$$\underset{p_{T}}{\max}\pi_{M}=D_{T}[(1-\lambda )p_{T}-c_{M}]$$ (4)

Then, the optimization problem of platform *T* can be formulated by:

$$\underset{d_{T}}{\max}\pi_{T}=\lambda p_{T}D_{T}-I_{T}(d_{T})$$ (5)

We adopt backward induction to solve the Stackelberg game; the Lemma 1 is obtained.

#### Lemma 1 (Response strategies of third-party platforms)

When *p*_*S*_ and *d*_*S*_ are given by the self-operated e-commerce platform, the optimal responses of the third-party e-commerce platform system with respect to DDS level and product retail price on this platform are:

$$\left\{\begin{array}{l} p_{T}(p_{S},d_{S})=\frac{2kt(p_{S}-hd_{S}+t)}{4kt-\lambda h^{2}}+\frac{c_{M}}{2(1-\lambda )}\\ d_{T}(p_{S},d_{S})=\frac{\lambda h(p_{S}+t-hd_{S})}{4kt-\lambda h^{2}} \end{array}\right.$$ (6)

All the proofs are shown in the S1 Appendix.

Lemma 1 shows that when the third-party e-commerce platform observes (or estimates through analysis) the information of the self-operated e-commerce platform’s product retail price and DDS level, it will make corresponding countermeasures according to the above response functions.

Back to Stage 1, we can solve the horizontal game model. First, the optimization problem of platform *S* can be formulated by:

$$\underset{p_{S},d_{S}}{\max}\pi_{S}=(p_{S}-c_{S})D_{S}-I_{S}(d_{S})$$ (7)

Solving this optimization problem leads to Lemma 2.

#### Lemma 2 (Response strategies of self-operated platforms)

When *p*_*T*_ and *d*_*T*_ are given by the third-party e-commerce platform, the optimal responses of the retail price and DDS level of the self-operated e-commerce platform are:

$$\left\{\begin{array}{l} p_{S}(p_{T},d_{T})=\frac{2kt(p_{T}-hd_{T}+c_{S}+t)-c_{S}h^{2}}{4kt-h^{2}}\\ d_{S}(p_{T},d_{T})=\frac{h(p_{T}-hd_{T}-c_{S}+t)}{4kt-h^{2}} \end{array}\right.$$ (8)

Similarly, Lemma 2 gives the response decisions that the self-operated e-commerce platform will make after mastering the information of the third-party e-commerce platform system’s retail price and DDS level.

Combining Lemmas 1 and 2, the correlation between the product retail price and DDS level of one platform and the response function of the other platform can be easily obtained by calculating the first derivatives of their response functions. For this, we have Property 1.

#### Property 1

Retail price *p*_*i*_ on e-commerce platform *i* and DDS level *d*_*i*_ of platform *i* are positively correlated with the price *p*_-*i*_ of its competitor and negatively correlated with the DDS level *d*_-*i*_ of its competitor.

Property 1 reveals that e-commerce platforms can make corresponding countermeasures to maximize their profits according to competitors’ strategies. E-commerce platforms can take raising the product price or improving the DDS level as the response strategy when their competitors’ product price rises. When the competitors’ DDS level increases, lowering the product price or reducing DDS investment is a reasonable response strategy.

Furthermore, we can obtain the optimal decision of the horizontal game in equilibrium. Since the actions of the horizontal game take place simultaneously, we combine the two sets of response functions in Lemmas 1 and 2 to obtain the equilibrium decisions of each stakeholder, which are shown in Theorem 1.

#### Theorem 1

Optimal decisions for each game member within both platform systems are:

1. The optimal decision for the self-operated e-commerce platform:
$$\left\{\begin{array}{l} d_{S}^{*}=\frac{h[\lambda h^{2}+k(c_{S}-3t)]}{k[h^{2}(1+\lambda )-6kt]}+\frac{hc_{M}(\lambda h^{2}-4kt)}{4kt[h^{2}(1-\lambda^{2})-6kt(1-\lambda )]}\\ p_{S}^{*}=\frac{c_{S}[h^{2}(1+\lambda )-4kt]+2t(\lambda h^{2}-3kt)}{h^{2}(1+\lambda )-6kt}+\frac{c_{M}(\lambda h^{2}-4kt)}{2[h^{2}(1+\lambda )-6kt]} \end{array}\right.$$ (9)
2. The optimal decision for the third-party e-commerce platform:
$$d_{T}^{*}=\frac{\lambda h[h^{2}-k(c_{S}+3t)]}{k[h^{2}(1+\lambda )-6kt]}+\frac{\lambda hc_{M}(h^{2}-2kt)}{4kt[h^{2}(1-\lambda^{2})-6kt(1-\lambda )]}$$ (10)
3. The optimal decision of the merchant on the third-party e-commerce platform:
$$p_{T}^{*}=\frac{2t[h^{2}-k(c_{S}+3t)]}{h^{2}(1+\lambda )-6kt}+\frac{c_{M}[h^{2}(2+\lambda )-8kt]}{2[h^{2}(1-\lambda^{2})-6kt(1-\lambda )]}$$ (11)

According to Theorem 1, Corollary 1 can be obtained by judging the positivity and negativity of $d_{i}^{*}$-value.

#### Corollary 1

A summary of the implementation of DDS strategies on self-operated and third-party e-commerce platforms in game equilibrium is shown in Table 2.

**Table 2:** DDS scenarios and corresponding conditions.

Conditions for implementing DDS	DDS scenarios in the market
*A*>0, *B*>0, *C*>0 or *A*<0, *B*<0, *C*<0	Both self-operated and third-party platforms that implement DDS strategies ($d_{S}^{*}>0$,$d_{T}^{*}>0$)
*A*>0, *B*≤0, *C*>0 or *A*<0, *B*≥0, *C*<0	The only self-operated platform that implements the DDS strategy ($d_{S}^{*}>0$,$d_{T}^{*}=0$)
*A*≥0, *B*<0, *C*<0 or *A*≤0, *B*>0, *C*>0	The only third-party platform that implements the DDS strategy ($d_{S}^{*}=0$,$d_{T}^{*}>0$)
*A*≥0, *B*≥0, *C*<0 or *A*≤0, *B*≤0, *C*>0	Neither self-operated nor third-party platforms that implement DDS strategies ($d_{S}^{*}=0$,$d_{T}^{*}=0$)

Note: *A* = 4*t*(1 - *λ*)[*λh*^2^ + *k*(*c*_*S*_ - 3*t*)] + *c*_*M*_ (*λh*^2^ - 4*kt*), *B*=4*t*(1 - *λ*)[*h*^2^ - *k* (*c*_*S*_ + 3*t*)] + *c*_*M*_ (*h*^2^ - 2*kt*), *C* = *h*^2^ (1 + *λ*) - 6*kt*.

From Corollary 1, the conditions for implementing DDS strategies must meet specific parameters’ relationships. Otherwise, both platforms will have no implementation motivation. In addition, we obtain an important finding that is different from the existing literature: it is not always advantageous to implement DDS strategies for self-operated and third-party platforms, and it is somewhat better not to implement DDS strategies under certain conditions.

**Fig 2:**
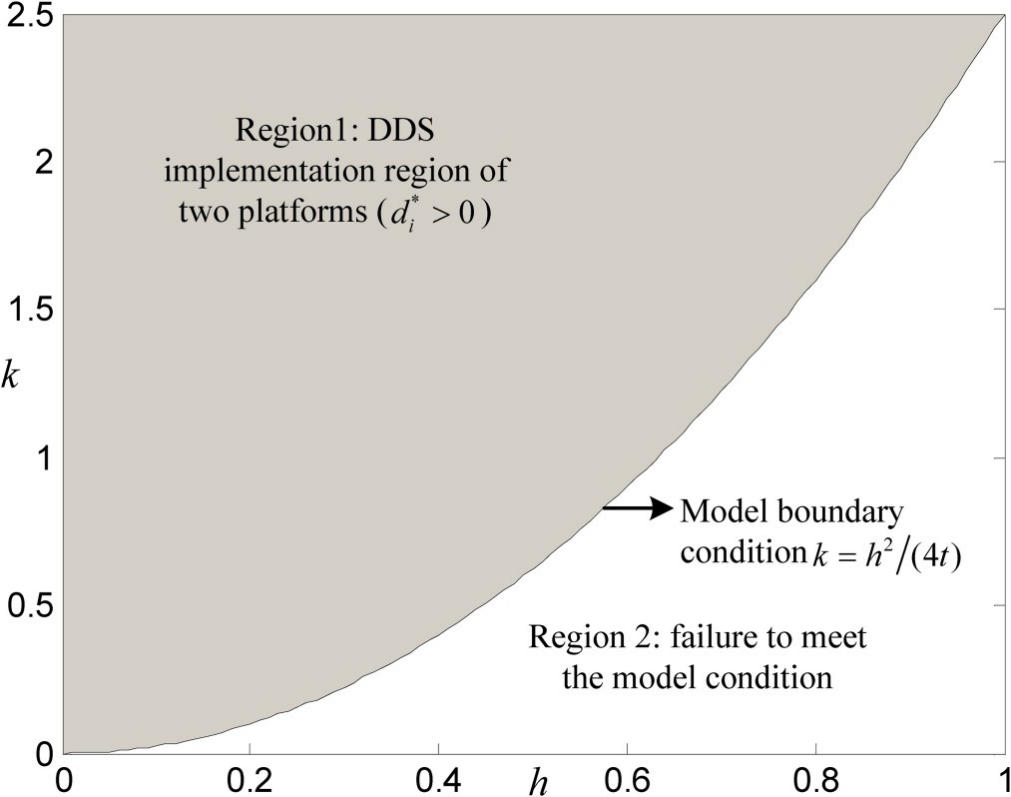
A feasible region of DDS implementation (with *k* and *h* change).

To show this more intuitively, we give a feasible region for DDS strategies implementation corresponding to a numerical case (*c*_*M*_ = 0.1, *c*_*S*_ = 0.2, *λ* = 3%, *t* = 0.1; this case is named Numerical Case 1), as shown in Fig 2. We noted that the DDS implementation region of two platforms (i.e., Region 1) in Fig 2 satisfies the condition *A*<0, *B*<0, and *C*<0 in Corollary 1. Certainly, Region 1 also meets the model condition $h\in (0,2\sqrt{kt})$. In this DDS implementation region, both self-operated and third-party platforms will prefer to implement DDS strategies. In addition, Fig 2 also illustrates the changing relationship between the influence coefficient *k* of DDS level on investment cost and the sensitivity coefficient *h* of DDS level on additional consumer utility when the DDS strategies are implemented. (we also consider the game of the two platforms without DDS strategies, and the equilibrium solutions of the reconstructed game model are $p_{S}^{*}=t+2c_{S}/3+c_{M}/\left[3(1-\lambda )\right]$ and $p_{T}^{*}=t+c_{S}/3+2c_{M}/\left[3(1-\lambda )\right]$). Judging from the region size of Regions 1 and 2, we can conclude that both platforms are more likely to implement DDS strategies than not, which logically means with a probability greater than 50%.

Moreover, by comparing the size relationship between the equilibrium solutions shown in Theorem 1, Proposition 1 can be obtained.

#### Proposition 1

There exist the following relationships between both e-commerce platforms with respect to DDS level and product retail price, respectively:

1. The relationship between the DDS levels of both platforms:
  1. $d_{S}^{*}>d_{T}^{*}$, if $\frac{2c_{S}(1-\lambda^{2})-6t{\left(1-\lambda \right)}^{2}-c_{M}(2-\lambda )}{h^{2}(1+\lambda )-6kt}>0$;
  2. $d_{S}^{*}<d_{T}^{*}$, if $\frac{2c_{S}(1-\lambda^{2})-6t{\left(1-\lambda \right)}^{2}-c_{M}(2-\lambda )}{h^{2}(1+\lambda )-6kt}<0$;
  3. $d_{S}^{*}=d_{T}^{*}$, if $2c_{S}(1-\lambda^{2})-6t{\left(1-\lambda \right)}^{2}-c_{M}(2-\lambda )=0$.
2. The relationship between the product retail prices of both platforms:
  1. $p_{S}^{*}>p_{T}^{*}$, if $\frac{c_{S}(1-\lambda )[h^{2}(1+\lambda )-2kt]-2th^{2}{\left(1-\lambda \right)}^{2}+c_{M}(2kt-h^{2})}{h^{2}(1+\lambda )-6kt}>0$;
  2. $p_{S}^{*}<p_{T}^{*}$, if $\frac{c_{S}(1-\lambda )[h^{2}(1+\lambda )-2kt]-2th^{2}{\left(1-\lambda \right)}^{2}+c_{M}(2kt-h^{2})}{h^{2}(1+\lambda )-6kt}<0$;
  3. $p_{S}^{*}=p_{T}^{*}$, if $c_{S}(1-\lambda )[h^{2}(1+\lambda )-2kt]-2th^{2}{\left(1-\lambda \right)}^{2}+c_{M}(2kt-h^{2})=0$.

Proposition 1 gives the dominant conditions of DDS levels for both platforms, and the dominance pattern will be changed when the values of related parameters are changed. Proposition 1 indicates that in the competition between both platforms, there is no situation where the level of DDS is constantly higher than the other, which shows that the DDS level is not limited to the platform mode, that is, the dominance of the DDS level has nothing to do with the operational modes of self-operated and third-party. Still, it will produce different results with the change in conditions. It is easy to get $d_{S}^{*}>d_{T}^{*}$ and $p_{S}^{*}>p_{T}^{*}$ by substituting Numerical Case 1 into Proposition 1. In other words, the self-operated platform’s DDS level is dominant, and its product retail price is higher under this Numerical Case 1.

## 5. Platform systems performance

In this section, we aim to compare and analyze the impacts of DDS and pricing strategies on the system’s performance of both platforms under duopoly competition. Both competing platforms dominate a platform system (as shown in Fig 1). Both platforms have evolved into a competition between their respective platform systems [47]. From the perspective of platform ecosystems, the value of e-commerce platform systems is jointly created by all members, including the leader (the e-commerce platform) and other participants on the platform [48]. Therefore, the system performance of the self-operated e-commerce platform considered in this paper is created by platform *S* and consumers on platform *S* (in the self-operated mode, the merchant is not the decision-making subject of the model. Thus, the system participants do not include it), the system performance of the third-party e-commerce platform is created by platform *T*, the merchant and consumers on platform *T* (in the third-party mode, the merchant is the decision-making subject of the model. Thus, the system participants include it). In order to facilitate the construction and analysis of the quantitative model in this paper, the platform system here only considers the platform, the merchant, and the consumers. From the perspective of the e-commerce platform ecosystem, it includes more subjects, such as logistics providers and financial services providers on the platform.

Referring to the method adopted by [49] in expressing the social welfare function of a system, we extend it to the performance evaluation of the e-commerce platform system. Therefore, the system performance *U*_*i*_ can be defined as the sum of all members’ welfare in this system; that is, the system performance of self-operated and third-party platforms is expressed as:

$$\left\{\begin{array}{l} U_{S}=\pi_{S}+CW_{S}\\ U_{T}=\pi_{T}+\pi_{M}+CW_{T} \end{array}\right.$$ (12)

where, the consumer welfare on platform *S* and platform *T* is the sum of consumer utility on the left and right of the indifference point *x*_0_, respectively. Combining Eq (2), we can obtain consumer welfare on both platforms $CW_{S}=\int_{0}^{x_{0}}u_{S}dx$ and $CW_{T}=\int_{x_{0}}^{1}u_{T}dx$ as follows:

$$\left\{\begin{array}{l} CW_{S}=\frac{\left(v-p_{S}+hd_{S})[p_{T}-p_{S}+t+h(d_{S}-d_{T})\right]}{2t}-\frac{{\left[p_{T}-p_{S}+t+h(d_{S}-d_{T})\right]}^{2}}{8t}\\ CW_{T}=v-p_{T}+hd_{T}-\frac{(v-p_{T}-t+hd_{T})[p_{T}-p_{S}+t+h(d_{S}-d_{T})]+t^{2}}{2t}-{\frac{\left[p_{T}-p_{S}+t+h(d_{S}-d_{T})\right]}{8t}}^{2} \end{array}\right.$$ (13)

Further, the system performance *U*_*i*_ of both platforms is obtained as follows:

$$\left\{\begin{array}{l} U_{S}=\frac{(v+hd_{S}-c_{S})[p_{T}-p_{S}+t+h(d_{S}-d_{T})]-ktd_{S}{}^{2}}{2t}-\frac{{\left[p_{T}-p_{S}+t+h(d_{S}-d_{T})\right]}^{2}}{8t}\\ U_{T}=\frac{(v+hd_{T}-c_{M})[p_{S}-p_{T}+t-h(d_{S}-d_{T})]-ktd_{T}{}^{2}}{2t}-\frac{{\left[p_{T}-p_{S}+t+h(d_{S}-d_{T})\right]}^{2}}{8t}+\frac{p_{T}-p_{S}+h(d_{S}-d_{T})}{2} \end{array}\right.$$ (14)

According to Eqs (13) and (14), Property 2 can be obtained by analyzing the correlation between the functions and the decision variables.

### Property 2

The correlations between consumer welfare on both platforms, the system performance of both platforms, and platforms’ decision-making are summarized in Table 3.

**Table 3:** The correlations between *C*W_i_, U_i_ and d_i_, p_i_.

Decision-making	Consumer welfare		System performance	
	*CW*_*S*_	*CW*_*T*_	*U*_*S*_	*U*_*T*_
*d*_*S*_	+	–	±	–
*d*_*T*_	–	+	–	±
*p*_*S*_	–	+	–	+
*p*_*T*_	+	–	+	–

Note: “+”, “–”, “±” indicate positive, negative, positive or negative correlation, respectively.

Property 2 reveals how the DDS level and product retail price decisions affect consumer welfare on both platforms and platform systems performance. According to Table 3, the increase in one platform’s retail price leads to a decrease in consumer welfare on its platform and an increase in consumer welfare on the other platform; on the contrary, when one platform’s DDS level is improved, it will contribute to increased consumer welfare on its platform and reduce consumer welfare on the competitor’s platform. Thus, consumer welfare on both platforms shows the law of “as one falls then another rises”, which plays a guiding role in constructing platform ecosystems. The platform can make corresponding decisions to win consumer welfare on its platform system under competition. In addition, there is the same relationship between the product price and the platform system performance; that is, one platform can improve its system performance and reduce the competitor’s system performance by adopting a price reduction strategy.

Interestingly, from the system performance perspective, “harming others may not benefit itself” if one platform consistently improves its DDS level. When the platform vigorously implements the DDS strategy, and the DDS level is improved, the competitor’s system performance will decline; however, its platform system performance may not increase. Although the improvement of the DDS level increases the platform’s consumer welfare, its system performance may decline, which indicates that this platform’s profit has decreased. Note that this phenomenon reflects that the industrial technology of big data is not mature currently, and the investment cost of the DDS strategy is too high.

**Fig 3:**
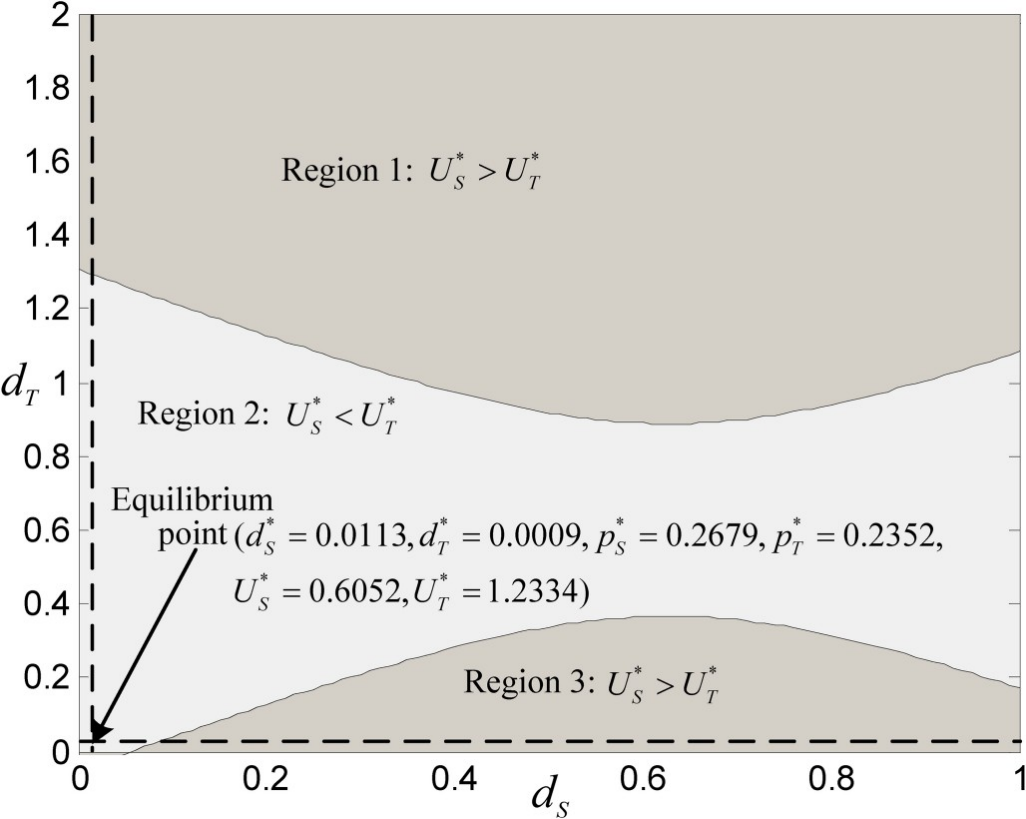
Comparison of systems performance between both platforms (with *d*_S_ and *d*_T_ change).

In order to compare the system performance of both platforms and discuss the relationship between platform systems performance and the DDS level of both platforms, we use the Numerical Case 2 (*c*_*M*_ = 0.1, *c*_*S*_ = 0.2, *λ* = 3%, *t* = 0.1, *v* = 2, *p*_*S*_ = *p*_*T*_ = 1, *h* = 0.05, *k* = 1.5) for simulation, and the result is shown in Fig 3. Note that Numerical Case 2 added the latter five parameters based on Numerical Case 1 in Section 4. Fig 3 shows the impact of DDS level combination changes of both platforms on the performance of their systems.

Fig 3 shows that when the game between both platforms is not in equilibrium, in most cases, the system performance of the self-operated platform is higher than that of the third-party platform. However, it is worth noting that when the DDS level of a third-party platform tends to the medium level, its platform system performance is often higher than that of a self-operated platform. In contrast, when it tends to the high or low level, its platform system performance is often lower than that of the self-operated platform. Further, when both platforms finally reach the game equilibrium, as shown in Fig 3, the equilibrium point ($d_{S}^{*}=0.0113,d_{T}^{*}=0.0009$) is relatively low, which reveals that the competitive environment under Numerical Case 2 makes the DDS levels of both platforms not too high.

## 6. Comparative analysis based on a numerical case

This section further compares and analyzes the impacts of key parameters *k* and *h* on DDS level, consumer welfare, platforms’ profit, and systems performance of both platforms through a numerical case (*c*_*M*_ = 0.1, *c*_*S*_ = 0.2, *λ* = 3%, *t* = 0.1, *v* = 2), which is derived from Numerical Case 2 in Section 5, so that the values of relevant parameters in the whole paper are consistent; indeed, the numerical case in this section ensures that DDS strategies are ready for implementation (see Region 1 of Fig 2).

### 6.1 Comparison of DDS levels

**Fig 4:**
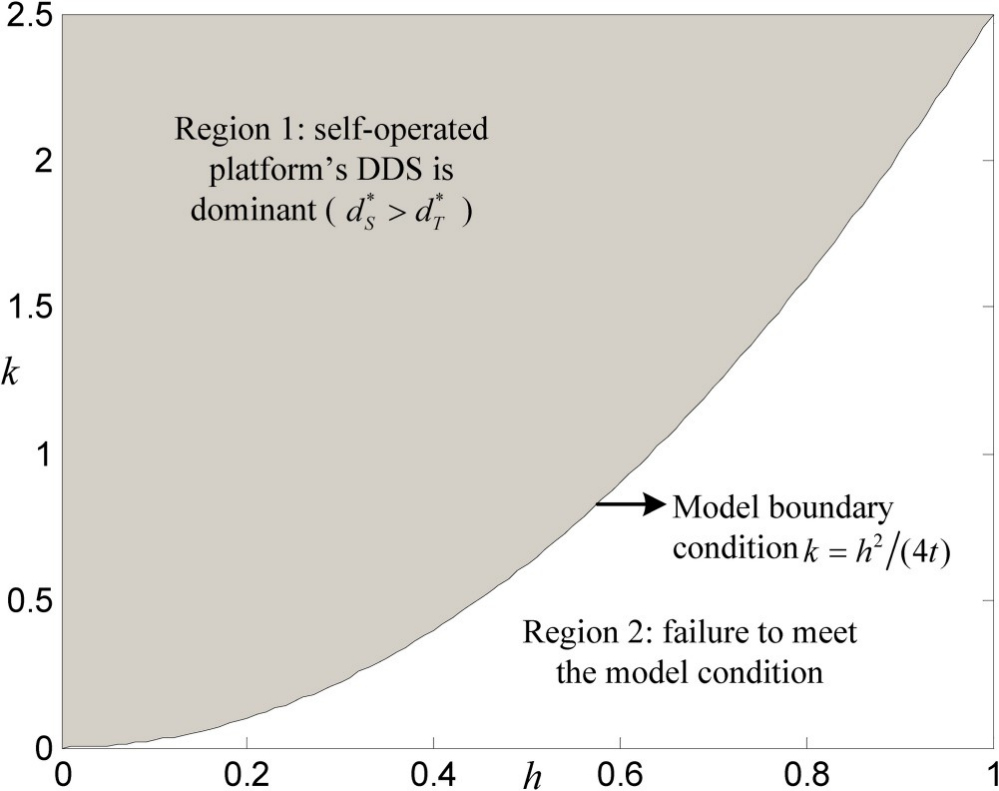
Comparison of DDS levels between both platforms.

Keeping the values of other parameters unchanged, the simulation result indicates that the combination of the influence coefficient *k* and the sensitivity coefficient *h* affects the dominance of DDS on both platforms, which is shown in Fig 4.

When *k* changes in the interval [0,2.5] and *h* changes in the interval [0,1], the DDS level of platform *S* is always higher than that of platform *T*, which shows that the self-operated platform’s DDS is dominant in all feasible regions (i.e., Region 1 of Fig 4), further, we have made some supplementary simulations through other groups of values, that is to be *h* fixed, and then to draw an impact graph of *k* on the DDS level of both platforms, which can be known: as the value of *k* decreases, the gap between the DDS level of third-party platform and that of the self-operated platform will gradually expand. In addition, by fixing the value of *k*, we also obtain that the advantage of the self-operated platform’s DDS level is growing with the increase of the *h* value. In general, in the face of the fact that the technology of the big data industry is becoming increasingly mature and consumers increasingly prefer high-quality service, the influence coefficient of DDS investment cost will gradually decrease, and the sensitivity of consumers to service quality will increase. The advantages of DDS in the future will be more prominent.

We must emphasize that the self-operated platform’s DDS under the numerical case in this section is dominant. The result is consistent with Proposition 1, but it does not mean that it is dominant under any conditions because it can be seen from Proposition 1 that there is a situation in which the third-party platform’s DDS is dominant. It’s just that the actual conditions will evolve to the maturity of industry technology and consumers’ preference for service quality; therefore, it is more likely that the self-operated platform’s DDS will dominate in the future. Considering the limited space, this paper will no longer set a numerical case in which the third-party platform’s DDS is dominant. However, this does not affect the main conclusion of this paper.

### 6.2 Comparison of consumer welfare

**Fig 5:**
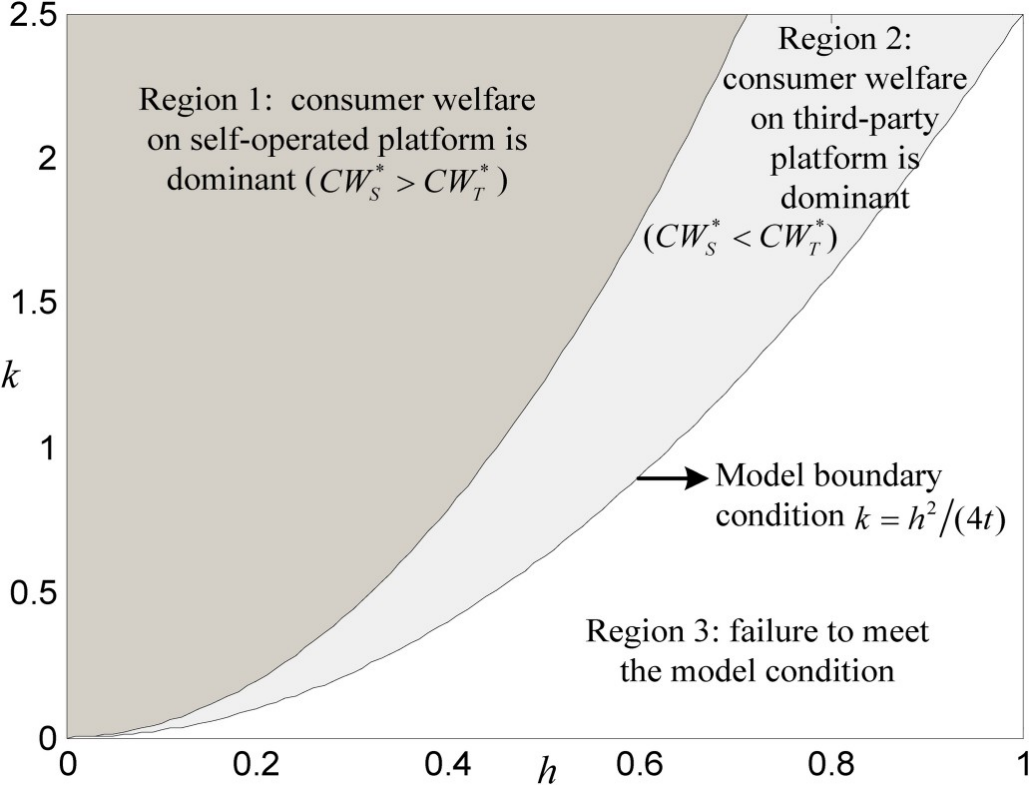
Comparison of consumer welfare between both platforms.

Keeping the values of other parameters unchanged, the impacts of the combination of *k* and *h* on consumer welfare on both platforms are shown in Fig 5. Note that Regions 1 and 2 are feasible regions for DDS strategies implementation. In Region 1, consumer welfare on the self-operated platform is higher than that on a third-party platform, and the opposite is true in Region 2. This shows that in Region 1, the consumer welfare on the self-operated platform is dominant, while the consumer welfare on the third-party platform is dominant in Region 2. Judging from the size of the respective dominant region, the situation in which consumer welfare is dominant on the self-operated is significantly more than that on the third-party platform.

It is noteworthy that, compared with Fig 4, although the self-operated platform’s DDS is dominant in both Regions 1 and 2 of Fig 5, consumer welfare is not always dominant in the two regions. In the DDS implementation region of both platforms (i.e., Regions 1 and 2 of Fig 5), when *k* is constant, Region 2 will be more likely to occur with the gradual increase of *h* value; at that time, consumer welfare on the third-party platform will be dominant. On the other hand, when *k* gradually decreases, and *h* is constant, Region 2 will also be more likely to appear, which means that as consumers become more sensitive to service quality and the technology of the big data industry gradually matures, the third-party platform will bring higher consumer welfare.

### 6.3 Comparison of profits and systems performance

**Fig 6:**
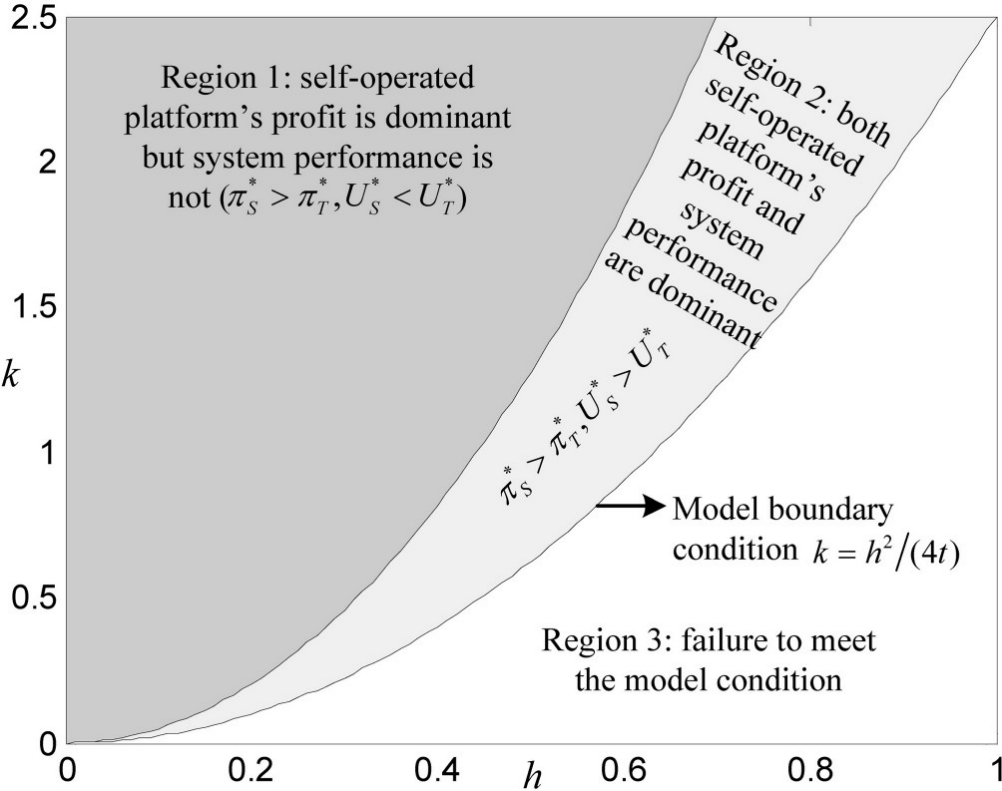
Comparison of profits and systems performance between both platforms.

Similarly, keeping the values of other parameters unchanged, the changes in both platforms’ profits and systems performance with the different combinations of *k* and *h* are shown in Fig 6. Since the two platforms aim at maximizing their respective profits in the competitive game, there will be a situation in which one platform’s profit is dominant, but its system performance is dominated.

As shown in Fig 6, the self-operated platform’s profits in Regions 1 and 2 are dominant; that is, in the DDS implementation region of both platforms, the self-operated platform always maintains a profit advantage and system performance is only dominant in Region 2. In contrast, its system performance is lower than that of the third-party platform in Region 1. It should be pointed out that, although the self-operated platform’s system performance does not have an advantage in Region 1, as consumers become more and more sensitive to service quality and the technology of big data is gradually mature, it is bound to show a trend of decrease in *k* and increase in *h*, and the realistic conditions will be closer to Region 2. By then, there will be a pattern in which the self-operated platform is dominant in both the profit and system performance.

Combining Figs 1–4, an interesting conclusion can be obtained: when the reality tends to be mature technology in the big data industry and consumers are more sensitive to service quality, the self-operated platform would be dominant in DDS level, profit, and system performance; however, the third-party platform would be dominant only in consumer welfare. Suppose we divide the large value of *k* and the small value of *h* into the early stage of the development of an e-commerce platform. In that case, the small value of *k* and the large value of *h* into the mature stage, and we assume that the early and mature stages belong to Regions 1 and 2, respectively, including Figs 5 and 6 (note that in Fig 4, there is no need to emphasize the regions corresponding to these two stages, since $d_{S}^{*}>d_{T}^{*}$ is always the case). Therefore, we can summarize the dominance of DDS, consumer welfare, profit, and system performance in the early and mature stages, as shown in Table 4.

**Table 4:** The dominance of platforms’ various indicators in the early and mature stages.

The stage	The self-operated platform				The third-party platform			
	DDS level	Consumer welfare	Profit	System performance	DDS level	Consumer welfare	Profit	System performance
**Early stage**	√	√	√	×	×	×	×	√
**Mature stage**	√	×	√	√	×	√	×	×

Note: “√” indicates it is dominant, “×” indicates it is dominated.

Table 4 intuitively shows that in the early stage of the development of an e-commerce platform, the self-operated platform has the advantages of DDS level, consumer welfare, and profit. The third-party platform has only the advantage of system performance. In the mature stage, the self-operated platform has DDS level, profit, and system performance advantages. The third-party platform has only the advantage of consumer welfare.

## 7. Discussion and conclusion

Data-driven operations management has become the focus of attention from all walks of life. For example, the e-commerce platform has the advantage of collecting large amounts of demand-side data, such as consumer clickstreams, transactions, and reviews. Applying big data and other technologies can give full play to the value of data. However, it is interesting to consider how e-commerce platforms develop DDS strategies in a competitive market and if e-commerce platform systems with various operating models have varied DDS strategy features. These questions gave us the idea for the investigation. The purpose of our study is to show the DDS investment decision behavior of self-operated and third-party e-commerce platforms and how DDS strategies affect platform ecosystem performance.

In this paper, we investigate the boundary condition for e-commerce platforms to implement DDS strategies under horizontal and vertical game structures. We further consider the impacts of the implementation of the DDS strategies on the system’s performance of their leading platforms. We find that the e-commerce platform has two options for responding when it observes that the price of its competitors’ products is rising: raising the product price or raising the DDS level. When a competitor’s DDS level is shown to have increased, one response option might be to lower the product’s price or cut back on DDS spending. A precondition must be satisfied before an e-commerce platform may implement a DDS strategy; otherwise, there would be no incentive to do so. In addition, the DDS level of either self-operated or third-party e-commerce platforms may be higher than each other in the face of various implementable conditions. The consumer welfare of both e-commerce platform systems demonstrates the law of “as one falls then another rises”. From the system performance perspective, when an e-commerce platform consistently improves its DDS level, it may appear that “harming others may not benefit oneself”. The benefits of the self-operated e-commerce platform system in terms of DDS level, profit, and system performance would become more noticeable as the big data technology sector, which is necessary for DDS, gradually matured and consumers’ preferences for high-quality services increased. However, the system of a third-party e-commerce platform would only demonstrate a benefit to consumer welfare.

This study’s contribution to academic literature is threefold. First, we quantitatively evaluate the performance of the platform ecosystem using microeconomic theory. This enriches the research literature on platform ecosystems (the existing literature is mainly empirical or case studies). Second, our study will expand the literature on platform-based operations management by examining price and service decisions in the presence of both horizontal and vertical games. Finally, this study well extends the data-driven management theory; we argue that data has become an essential resource in the competition of Internet enterprises.

The practical implications of this research can be directed mainly at e-commerce platforms and merchants. For e-commerce platforms, they might use our conclusions as a guide when creating a platform ecosystem. Moreover, they can also develop DDS strategies based on our findings. For merchants, their practical insights lie in how to set prices and play the role of a good ecosystem member. The significant observation is that the platform, the dominant player in the ecosystem, makes pricing or service decisions that affect platform profits, consumer welfare, and ecosystem performance.

Our research has several limitations in the model assumptions, such as setting the consumer’s preference cost for both platforms to the same value. But in fact, the cost of preference varies from consumer to consumer. In addition, this paper considers fewer participants in the e-commerce platform systems, while often many members in a platform ecosystem. Therefore, future research work would be extended to gain a deeper understanding of the role of DDS strategy in the digital platform ecosystem. The quantitative model of the platform ecosystem can be further characterized. It is our hope that this work will spark discussions and provide an impetus for further research on operational strategies that can achieve value co-creation in the digital platform ecosystem.

## Supporting information

S1 Appendix (DOC)
