# Supplementary material for: The role of data-driven services strategy in platform competition: A system performance perspective
Source: PLoS One. 2023 Jan 26;18(1):e0272547. doi: 10.1371/journal.pone.0272547 (PMC9879479; doi:10.1371/journal.pone.0272547)
Supplement: S1 Appendix — (DOC) [file pone.0272547.s001.doc]

**Appendix**

**Proof of Lemma 1**

Since $\frac{\partial^{2}\pi_{M}}{\partial p_{T}^{2}}=-\frac{1-\lambda}{t}<0$, we can see that $\pi_{M}$ is the concave function with respect to $p_{T}$. Taking the first-order condition $\frac{\partial\pi_{M}}{\partial p_{T}}=0$, we have $p_{T}=\frac{(1-\lambda)p_{S}-(1-\lambda)[h(d_{S}-d_{T})-t]+c_{M}}{2(1-\lambda)}$, substituting into the objective function $\pi_{T}$ yields $\frac{\partial^{2}\pi_{T}}{\partial d_{T}^{2}}=\frac{\lambda h^{2}-4kt}{4t}$. Since $h\in(0,2\sqrt{kt})$ and obviously the commission rate $\lambda\in(0,1)$ holds. Therefore, $\frac{\partial^{2}\pi_{T}}{\partial d_{T}^{2}}<0$, that is, $\pi_{T}$ is the concave function with respect to $d_{T}$. Taking the first-order condition $\frac{\partial\pi_{T}}{\partial d_{T}}=0$, we get $d_{T}(p_{S},d_{S})=\frac{\lambda h(p_{S}+t-hd_{S})}{4kt-\lambda h^{2}}$, substituting into $p_{T}$, then we have $p_{T}(p_{S},d_{S})=\frac{2kt(p_{S}-hd_{S}+t)}{4kt-\lambda h^{2}}+\frac{c_{M}}{2(1-\lambda)}$. The proof is completed.

**Proof of Lemma 2**

According to the objective function $\pi_{S}$, the Hessian matrix is obtained as:

| $H\text{=}\left(\begin{matrix} -\frac{1}{t} & \frac{h}{2t} \\ \frac{h}{2t} & -k \end{matrix}\right)$ | (A1) |
| --- | --- |

Its first-order and second-order sequential principal minors are $\left|H_{1}\right|\text{=}-\frac{1}{t}<0$ and $\left|H_{2}\right|\text{=}\frac{4kt-h^{2}}{4t^{2}}$, respectively. Also, according to the model condition $h\in(0,2\sqrt{kt})$ considered in Section 3, we have . Thus, $\pi_{S}$ is a joint concave function with respect to $p_{S}$ and $d_{S}$, there exists a unique optimal solution, which can be obtained from the first-order conditions $\frac{\partial\pi_{S}}{\partial p_{S}}=0$ and $\frac{\partial\pi_{S}}{\partial d_{S}}=0$:

| $\left\{\begin{matrix} p_{S}(p_{T},d_{T})=\frac{2kt(p_{T}-hd_{T}+c_{S}+t)-c_{S}h^{2}}{4kt-h^{2}} \\ d_{S}(p_{T},d_{T})=\frac{h(p_{T}-hd_{T}-c_{S}+t)}{4kt-h^{2}} \end{matrix}\right.$ | (A2) |
| --- | --- |

The optimal response functions of platform *S* with respect to $p_{T}$ and $d_{T}$ given by platform *T* are shown in Eqs. (A2). The proof is completed.

**Proof of Property 1**

Finding the first-order partial derivative of the reaction function $p_{T}(p_{S},d_{S})$ with respect to $p_{T}$ in Lemma 1 yields $\frac{\partial p_{S}(p_{T},d_{T})}{\partial p_{T}}=\frac{2kt}{4kt-h^{2}}>0$. Similarly, by taking the first-order partial derivatives of the other reaction functions in Lemma 1 and Lemma 2, we can easily draw conclusions. The proof is completed.

**Proof of Theorem 1**

Based on the reaction functions in Lemmas 1 and 2, the simultaneous equations are as follows:

| $\left\{\begin{matrix} \frac{2kt(p_{T}-hd_{T}+c_{S}+t)-c_{S}h^{2}}{4kt-h^{2}}-p_{S}=0 \\ \frac{h(p_{T}-hd_{T}-c_{S}+t)}{4kt-h^{2}}-d_{S}=0 \\ \frac{2kt(p_{S}-hd_{S}+t)}{4kt-\lambda h^{2}}+\frac{c_{M}}{2(1-\lambda)}-p_{T}=0 \\ \frac{\lambda h(p_{S}+t-hd_{S})}{4kt-\lambda h^{2}}-d_{T}=0 \end{matrix}\right.$ | (A3) |
| --- | --- |

$d_{S}^{*}$, $p_{S}^{*}$, $d_{T}^{*}$, $p_{T}^{*}$ can be obtained by solving the above equations. The proof is completed.

**Proof of Corollary 1**

$d_{i}^{*}>0$ is a necessary condition for the implementation of DDS strategy. Otherwise, the optimal solution of the platform profit function with respect to DDS level in the equilibrium state is obtained at the boundary $d_{i}=0$, which means that the platform does not implement DDS strategy. By Theorem 1, let $d_{S}>0$, we have $\left\{\begin{matrix} A>0 \\ C>0 \end{matrix}\right.$ or $\left\{\begin{matrix} A<0 \\ C<0 \end{matrix}\right.$; let $d_{S}\leq0$, we have $\left\{\begin{matrix} A\geq0 \\ C<0 \end{matrix}\right.$ or $\left\{\begin{matrix} A\leq0 \\ C>0 \end{matrix}\right.$; let $d_{T}>0$, we have $\left\{\begin{matrix} B>0 \\ C>0 \end{matrix}\right.$ or $\left\{\begin{matrix} B<0 \\ C<0 \end{matrix}\right.$; let $d_{T}\leq0$, we have $\left\{\begin{matrix} B\geq0 \\ C<0 \end{matrix}\right.$ or $\left\{\begin{matrix} B\leq0 \\ C>0 \end{matrix}\right.$. Thus, the following four scenarios can be obtained:

(1) $d_{S}^{*}>0$ and $d_{T}^{*}>0$, if *A*>0, *B*>0, *C*>0 or *A*<0, *B*<0, *C*<0.

(2) $d_{S}^{*}>0$ and $d_{T}^{*}=0$, if *A*>0, *B*≤0, *C*>0 or *A*<0, *B*≥0, *C*<0.

(3) $d_{S}^{*}=0$ and $d_{T}^{*}>0$, if *A*≥0, *B*<0, *C*<0 or *A*≤0, *B*>0, *C*>0.

(4) $d_{S}^{*}=0$ and $d_{T}^{*}=0$, if *A*≥0, *B*≥0, *C*<0 or *A*≤0, *B*≤0, *C*>0.

The proof is completed.

**Proof of Proposition 1**

According to Theorem 1, we have $d_{S}^{*}-d_{T}^{*}=\frac{h[2c_{S}(1-\lambda^{2})-6t(1-\lambda)^{2}-c_{M}(2-\lambda)]}{2(1-\lambda)[h^{2}(1+\lambda)-6kt]}$. Since $1-\lambda>0$ and $h>0$, therefore, the following three scenarios exist:

(1) $d_{S}^{*}>d_{T}^{*}$, if $\frac{2c_{S}(1-\lambda^{2})-6t(1-\lambda)^{2}-c_{M}(2-\lambda)}{h^{2}(1+\lambda)-6kt}>0$.

(2) $d_{S}^{*}<d_{T}^{*}$, if $\frac{2c_{S}(1-\lambda^{2})-6t(1-\lambda)^{2}-c_{M}(2-\lambda)}{h^{2}(1+\lambda)-6kt}<0$.

(3) $d_{S}^{*}=d_{T}^{*}$, if $2c_{S}(1-\lambda^{2})-6t(1-\lambda)^{2}-c_{M}(2-\lambda)=0$.

In addition, $p_{S}^{*}-p_{T}^{*}=\frac{c_{S}(1-\lambda)[h^{2}(1+\lambda)-2kt]-2th^{2}(1-\lambda)^{2}+c_{M}(2kt-h^{2})}{(1-\lambda)[h^{2}(1+\lambda)-6kt]}$, Similar analysis can draw conclusions. The proof is completed.

**Proof of Property 2**

From Eq. (13), $\frac{\partial U_{T}}{\partial p_{T}}=\frac{(v-p_{S}-t+hd_{S})-3(v-p_{T}+hd_{T})}{4t}=\frac{u_{S}\left|_{x=1}\right.-3u_{T}\left|_{x=1}\right.}{4t}$. When $x=1$, obviously $u_{S}<u_{T}$ (otherwise, the market is monopolized by platform *S*, which is inconsistent with the model background). Therefore, we have $\frac{\partial U_{T}}{\partial p_{T}}<0$. Since $\frac{\partial U_{T}}{\partial d_{T}}=\frac{h(3u_{T}\left|_{x=1}\right.-u_{S}\left|_{x=1})\right.}{4t}>0$, similarly, it is easy to get $\frac{\partial U_{T}}{\partial p_{S}}>0$, $\frac{\partial U_{T}}{\partial d_{S}}<0$, $\frac{\partial U_{S}}{\partial p_{S}}<0$, $\frac{\partial U_{S}}{\partial p_{T}}>0$, $\frac{\partial U_{S}}{\partial d_{S}}>0$, $\frac{\partial U_{S}}{\partial d_{T}}<0$.

According to Eq. (14), we have $\frac{\partial P_{S}^{PE}}{\partial p_{S}}=-\frac{u_{S}\left|_{x=1}\right.+u_{T}\left|_{x=1}+2(p_{S}-c_{S})\right.}{4t}$, obviously $\frac{\partial P_{S}^{PE}}{\partial p_{S}}<0$. Furthermore, $\frac{\partial^{2}P_{S}^{PE}}{\partial d_{S}^{2}}=\frac{3h^{2}-4kt}{4t}$, since $h\in(0,2\sqrt{kt})$, thus the positivity and negativity of $\frac{\partial^{2}P_{S}^{PE}}{\partial d_{S}^{2}}$ cannot be determined; that is, $P_{S}^{PE}$ may be a concave or convex function with respect to $d_{S}$, and the positive or negative of $\frac{\partial P_{S}^{PE}}{\partial d_{S}}=\frac{h[2v+p_{T}-p_{S}+t+h(3d_{S}-d_{T})-2c_{S}]-4ktd_{S}}{4t}$ cannot be determined either. Therefore, $P_{S}^{PE}$ and $d_{S}$ may be positively or negatively correlated. Similarly, we can obtain that $P_{T}^{PE}$ and $d_{T}$ may be positively or negatively correlated. Besides, it is easy to prove that the following holds $\frac{\partial P_{S}^{PE}}{\partial p_{T}}>0$, $\frac{\partial P_{S}^{PE}}{\partial d_{T}}<0$, $\frac{\partial P_{T}^{PE}}{\partial p_{S}}>0$, $\frac{\partial P_{T}^{PE}}{\partial p_{T}}<0$, $\frac{\partial P_{T}^{PE}}{\partial d_{S}}<0$. The proof is completed.
